# The Role of Artificial Intelligence on Tumor Boards: Perspectives from Surgeons, Medical Oncologists and Radiation Oncologists

**DOI:** 10.3390/curroncol31090369

**Published:** 2024-08-27

**Authors:** Valerio Nardone, Federica Marmorino, Marco Maria Germani, Natalia Cichowska-Cwalińska, Vittorio Salvatore Menditti, Paolo Gallo, Vittorio Studiale, Ada Taravella, Matteo Landi, Alfonso Reginelli, Salvatore Cappabianca, Sergii Girnyi, Tomasz Cwalinski, Virginia Boccardi, Aman Goyal, Jaroslaw Skokowski, Rodolfo J. Oviedo, Adel Abou-Mrad, Luigi Marano

**Affiliations:** 1 Department of Precision Medicine, University of Campania “L. Vanvitelli”, 80131 Naples, Italy; valerio.nardone@unicampania.it (V.N.); vittoriosalvatore.menditti@studenti.unicampania.it (V.S.M.); paolo.gallo@studenti.unicampania.it (P.G.); alfonso.reginelli@unicampania.it (A.R.); salvatore.cappabianca@unicampania.it (S.C.); 2 Unit of Medical Oncology 2, Azienda Ospedaliera Universitaria Pisana, 56126 Pisa, Italy; f.marmorino@studenti.unipi.it (F.M.); m.germani@studenti.unipi.it (M.M.G.); v.studiale@studenti.unipi.it (V.S.); a.taravella1@studenti.unipi.it (A.T.); m.landi31@studenti.unipi.it (M.L.); 3 Department of Translational Research and New Technologies in Medicine and Surgery, University of Pisa, 56126 Pisa, Italy; 4 Department of Oncology and Radiotherapy, Medical University of Gdańsk, 80-210 Gdańsk, Poland; nat.cichowska@gumed.edu.pl; 5 Department of General Surgery and Surgical Oncology, “Saint Wojciech” Hospital, “Nicolaus Copernicus” Health Center, 80-462 Gdańsk, Poland; sgirnyi@copernicus.gda.pl (S.G.); tcwalinski@copernicus.gda.pl (T.C.); j.skokowski@amisns.edu.pl (J.S.); l.marano@amisns.edu.pl (L.M.); 6 Division of Gerontology and Geriatrics, Department of Medicine and Surgery, University of Perugia, 06123 Perugia, Italy; virginia.boccardi@unipg.it; 7 Adesh Institute of Medical Sciences and Research, Bathinda 151109, Punjab, India; doc.aman.goyal@gmail.com; 8 Department of Medicine, Academy of Applied Medical and Social Sciences-AMiSNS: Akademia Medycznych I Spolecznych Nauk Stosowanych, 82-300 Elbląg, Poland; 9 Nacogdoches Medical Center, Nacogdoches, TX 75965, USA; 10 Tilman J. Fertitta Family College of Medicine, University of Houston, Houston, TX 77021, USA; 11 College of Osteopathic Medicine, Sam Houston State University, Conroe, TX 77304, USA; 12 Centre Hospitalier Universitaire d’Orléans, 45100 Orléans, France; adel.abou-mrad@orange.fr

**Keywords:** artificial intelligence in oncology, multidisciplinary tumor boards, clinical decision support systems, personalized cancer treatment, data-driven oncology, AI-enhanced cancer care

## Abstract

The integration of multidisciplinary tumor boards (MTBs) is fundamental in delivering state-of-the-art cancer treatment, facilitating collaborative diagnosis and management by a diverse team of specialists. Despite the clear benefits in personalized patient care and improved outcomes, the increasing burden on MTBs due to rising cancer incidence and financial constraints necessitates innovative solutions. The advent of artificial intelligence (AI) in the medical field offers a promising avenue to support clinical decision-making. This review explores the perspectives of clinicians dedicated to the care of cancer patients—surgeons, medical oncologists, and radiation oncologists—on the application of AI within MTBs. Additionally, it examines the role of AI across various clinical specialties involved in cancer diagnosis and treatment. By analyzing both the potential and the challenges, this study underscores how AI can enhance multidisciplinary discussions and optimize treatment plans. The findings highlight the transformative role that AI may play in refining oncology care and sustaining the efficacy of MTBs amidst growing clinical demands.

## 1. Introduction

The established protocol to ensure state-of-the-art cancer treatment includes the use of multidisciplinary tumor boards (MTBs) [1]. The multidisciplinary approach to management (MDM) is progressively gaining a leading role, highlighted by the creation of tumor committees or MTBs meetings [2]. These formal meetings involve key experts who meet regularly, often on a weekly basis, to review and discuss the diagnosis and management of patients with cancer. Participation in such assemblies is typically reserved for a small group of professionals: medical oncologists, surgeons, radiologists, pathologists, radiation oncologists, and other specialists, in accordance with the nature and specialization of the oncology committee. MTBs meetings play a crucial role in refining diagnoses for patients suffering from malignant tumors: they contribute significantly to increase the likelihood that these patients will benefit from personalized care, allowing them to access the best treatment [3]. This procedure is based on the most modern and sophisticated scientific and medical knowledge, guaranteeing a cutting-edge approach to ensuring the well-being of patients. Currently, treatment choices are delegated to a MTBs which has shown an improvement in patient outcomes. However, recently, the MDT system in oncology care has experienced increasing strains, due to rising cancer incidence, the growing popularity of MDT work, and financial pressures [4].

The evolution of artificial intelligence (AI) in the medical field contributes to the progression of programs specifically designed to help doctors make diagnoses, make treatment decisions, and predict outcomes [5]. These systems are meticulously designed to provide substantial support to healthcare professionals in their daily activities, facilitating their engagement in tasks that include data manipulation and knowledge management. Among the technologies used are artificial neural networks (ANN), evolutionary computing and hybrid intelligent systems. In 2020, Lee K et al. analyzed the effectiveness of Watson for Oncology (WfO), an advanced clinical decision support system based on AI [6]. It should be noted that WfO does not play a direct role in the treatment of patients, but it offers to the medical staff essential tools to manage and monitor patients’ health, while also facilitating access to crucial medical information. The system thoroughly evaluates patients’ medical information against a broad spectrum of clinical evidence, such as medical journals, cancer treatment guidelines, drug information, and medical texts. The study mainly focused on assessing patient satisfaction and perception of the hospital. Of the 285 participants involved in the research, 45.3% (129 patients) underwent treatment by a Multidisciplinary Team using Watson for Oncology (MDT-WfO), while the remaining 54.7% (156 patients) was treated by a conventional MDT. In the MDT-WfO group, a notable rate of positive change in patient perception after treatment was observed, standing at 86.8%, while in the MDT group this rate was 71.2%. Increasingly widespread adoption highlights the growing importance of AI, including the fields of machine learning (ML) and deep learning (DL), in the context of clinical decision-making [7]. The widespread implementation of AI algorithms in healthcare could pave the way for clinically relevant information, revolutionizing the approach to patient classification, treatment development, disease study, and clinical decisions.

To our knowledge, no other study has conducted an analysis and review of the impact of AI on MTBs from a broad perspective encompassing multiple specialties and disciplines dedicated to the care of cancer patients in a multidisciplinary environment. The present study aimed to examine the possible impact of AI on surgical oncology, medical oncology, and radiotherapy perspectives in their specialties and sub-specialties within MTBs.

## 2. Surgeon Perspective

**Figure 1:**
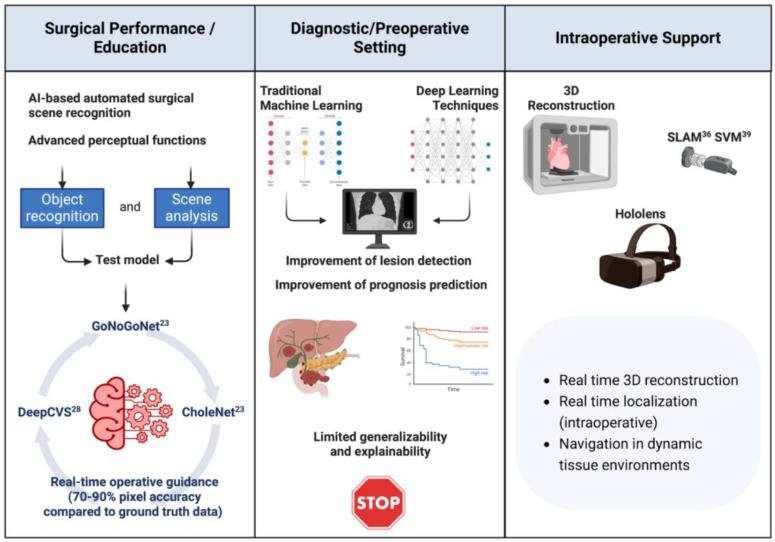
Application of artificial intelligence (AI) across different phases of surgical oncology, including surgical performance and education, diagnostic/preoperative settings, and intraoperative support. **Surgical Performance/Education**: AI-based automated surgical scene recognition enhances advanced perceptual functions such as object recognition and scene analysis. Systems like GoNoGoNet, DeepCVS, and CholeNet facilitate real-time operative guidance with a 70–90% pixel accuracy compared to ground truth data. **Diagnostic/Preoperative Setting**: Traditional machine learning and deep learning techniques improve lesion detection and prognosis prediction. While these techniques enhance diagnostic capabilities, they face challenges related to limited generalizability and explainability. **Intraoperative Support**: Tools like 3D reconstruction, SLAM (Simultaneous Localization and Mapping), SVM (Support Vector Machine), and Hololens provide real-time 3D reconstruction, localization, and navigation in dynamic tissue environments, aiding surgeons during operations.

AI has gained significant traction in the surgical specialties in recent times. While Surgical Decision Support (SDS) and AI are distinct concepts, SDS can leverage AI techniques to enhance the delivery of surgical care. This enhancement may occur through direct patient interaction via diagnostic and therapeutic measures or by providing clinicians with data-driven insights into their own performance. Previous studies have explored the diagnostic and therapeutic potential of SDS and AI, especially with the main focus on their application in surgical education [8,9,10,11]. This includes decision support, coaching, feedback mechanisms, and performance assessment. The dissemination of surgical practice through technology has the potential to empower surgeons worldwide to enhance the quality of global surgical care. Research has consistently shown a correlation between surgical technique, skill, and patient outcomes [12,13]. Therefore, AI has the capacity to aggregate surgical experiences, akin to initiatives in genomics and biobanks, aiming to integrate decision-making capabilities and techniques from the global surgical community into every surgical procedure [14]. By utilizing the concept of big data, a “collective surgical consciousness” could be established, encapsulating the comprehensive knowledge of the field. This could pave the way for technology-driven real-time clinical decision support, such as intraoperative guidance similar to GPS navigation. The different applications of AI in surgery are summarized in Figure 1 and Table 1.

### 2.1. AI to Enhance Surgical Performance and Training

Over the past three decades, data have revealed alarmingly high rates of preventable adverse events among hospitalized surgical patients [15]. Root-cause analyzes often attribute these errors to events occurring during surgery, emphasizing errors in judgment or decision-making leading to adverse outcomes [16,17,18,19,20,21]. Cognitive skills are deemed crucial for developing elite surgeons, as supported by literature in surgical education highlighting the significance of intraoperative judgment and decision-making for surgical performance and outcomes [20,22,23]. For instance, errors in visual pattern recognition can result in misinterpreting surgical anatomy, leading to critical injuries during procedures like laparoscopic cholecystectomy [24]. The digitalization of the surgical field has enabled the collection of vast datasets from the operating room (OR), including images and videos, prompting interest in ML to augment surgical teams’ performance. Advances in computer vision, particularly DL, offer opportunities to develop algorithms capable of advanced perceptual functions, such as object recognition and scene analysis within surgical videos. Given that most errors stem from advanced cognitive skills, there is significant potential in developing algorithms to analyze surgical data and enhance decision-making. However, applying computer vision to surgery presents challenges. Anatomical structures are often poorly demarcated and obscured by tissues, complicating model training for intraoperative guidance. Additionally, surgical videos exhibit variability in quality and background noise, posing challenges for algorithm development. Moreover, there is considerable variation among experts in cognitive behaviors, hindering the establishment of a gold-standard reference for algorithm training and evaluation. To address these challenges, Madani A et al. [25] proposed the visual concordance test (VCT) to establish expert consensus within surgical fields.

Surgeons annotate frames from surgical videos, with annotations compiled to create a “heat map” indicating agreement among experts. Leveraging VCT, models like GoNoGoNet and CholeNet were developed to detect safe and dangerous areas during laparoscopic cholecystectomy, offering real-time guidance to surgeons [26]. These algorithms were designed to automatically identify and delineate safe dissection areas (referred to as “Go zones”), hazardous dissection areas (“No-Go zones”), and other anatomical structures during laparoscopic cholecystectomy. In this investigation, a dataset comprised of 290 laparoscopic cholecystectomy videos sourced from 136 institutions across 37 countries was utilized to train these algorithms, achieving over 90% pixel accuracy and substantial spatial overlap compared to ground truth data. The real-time overlay of Go and No-Go zones could offer feedback and guidance to surgeons seeking to acquire new skills, enhance their performance, or manage particularly challenging operations. Considering the promising outcomes of GoNoGoNet, its potential applications in surgical oncology are noteworthy. The primary objective of most cancer surgeries is to perform an adequate oncologic resection while minimizing perioperative morbidity. Deviating from the ideal dissection plane can result in either an oncologically inadequate resection or an increased risk of complications due to damage to surrounding structures. Several research groups are presently endeavoring to develop models that offer real-time guidance on the optimal dissection plane during cancer surgeries to mitigate early perioperative complications and enhance long-term oncologic outcomes. Surgical decision-making does not always pertain to a specific location within the surgical field (e.g., where to dissect); it often involves higher-level considerations regarding the tactical approach of the operation. Therefore, AI-based automated scene recognition and assessment could prove beneficial in critical decision points of procedures, particularly in scenarios with significant operator variability. For instance, during laparoscopic cholecystectomy, it is crucial not only to maintain dissection within a safe plane to minimize the risk of major bile duct injury but also to refrain from dividing cystic structures until achieving a critical view of safety (CVS) [27,28]. Given that the determination of CVS is highly dependent on the operator, a model offering decision support to surgeons in real-time regarding the most optimal strategy could be immensely advantageous [29,30]. Mascagni et al. [31] recently presented their findings on DeepCVS, a two-stage model for segmenting hepatocystic anatomy and predicting the achievement of each element of the CVS. The model demonstrated a mean average precision exceeding 70%, suggesting its potential to augment intraoperative judgment in challenging situations.

### 2.2. AI to Enhance Preoperative Setting

AI plays a pivotal role in preoperative settings, where surgeons plan surgical procedures based on patients’ medical records and imaging [32]. Common imaging modalities include X-ray, CT, ultrasound, and MRI, facilitating tasks such as anatomical classification, detection, segmentation, and registration. In classification tasks, AI determines the diagnostic value of medical images or organ/lesion volumes and dimensions. Traditional ML and image analysis methods are prevalent, but DL-based techniques are gaining prominence [33]. These methods typically employ convolutional layers to extract information and fully connected layers to assess diagnostic value. For instance, a classification pipeline utilizing Google’s Inception and ResNet architecture was proposed for segmenting lung, bladder, and breast cancers. DL models have been demonstrated to recognize intracranial hemorrhage, midline shift, and mass effect from head CT scans, improving prediction accuracy for patient outcomes after cardiosurgical care compared to conventional tools [34,35]. Detection involves spatially localizing regions of interest, often represented as bounding boxes or landmarks, and may include image- or region-level classification. DL approaches excel in detecting anomalies or medical conditions, employing convolutional layers for feature extraction and regression layers to determine bounding box properties. For example, a deeply stacked convolutional autoencoder was trained to detect prostate cancer from 4D positron-emission tomography images, while a 3D CNN with roto-translation group convolutions showed promising results in pulmonary nodule detection [36]. Deep reinforcement learning based on an extension of the deep Q-network was utilized to learn a search policy from dynamic contrast-enhanced MRI for breast lesion detection [37]. In addition to these advancements, AI optimization algorithms have emerged as critical tools in enhancing the accuracy and efficiency of preoperative planning. Algorithms such as the Enhanced Moth-Flame Optimizer and Enhanced Gaussian Bare-Bones Grasshopper Optimization have been effectively applied in the segmentation and diagnosis of tumors, which is essential for multidisciplinary treatment approaches [38,39]. These algorithms optimize feature selection and image segmentation, enabling more precise identification and classification of tumors, thereby supporting the formulation of effective treatment plans across various medical specialties [40]. Anyway, while DL-based methods show potential superiority over conventional approaches, they face challenges such as limited generalizability and explainability.

Collaboration between surgeons and AI researchers is essential to generate large-scale annotated datasets and develop techniques like meta-learning for improved performance. Disparities between medical and natural images may hinder clinical applicability, requiring exploration of transfer learning techniques. Additionally, integrating personalized patient data into AI development could enhance early detection and treatment options, minimizing surgical risks and recovery time.

### 2.3. AI for Intraoperative Support

Computer-assisted intraoperative guidance has long been fundamental to Minimally Invasive Surgery (MIS). Learning strategies have been extensively incorporated into the evolution of intraoperative guidance to offer improved visualization and localization during surgery that can be categorized into four primary areas: shape instantiation, endoscopic navigation, tissue tracking, and augmented reality [32]. For intraoperative 3D reconstruction, various imaging modalities such as MRI, CT, or ultrasound are employed to scan 3D volumes. However, this process can be time-consuming or yield scans with low resolution. Streamlining the number of images required for 3D shape reconstruction can enable real-time reconstruction of a surgical scene, while improved protocols can enhance reconstruction resolution. Real-time 3D shape instantiation from a limited amount of 2D images is an emerging research area [41]. For instance, researchers have instantiated a 3D prostate shape from multiple nonparallel 2D ultrasound images using a radial basis function [42]. Similarly, 3D shapes of stent grafts in different deployment states were instantiated from single 2D fluoroscopy projections using mathematical modeling and neural networks. Additionally, methods like equally weighted focal U-Net were proposed to automatically segment markers on stent grafts, enhancing intraoperative shape instantiation efficiency. Principal component analysis (PCA), statistical shape model (SSM), and partial least square regression (PLSR) were utilized to instantiate liver shapes from single 2D projections [35,43]. Recently, advanced DL strategies have been proposed for 3D shape instantiation from single 2D images. These advancements contribute to real-time and efficient intraoperative 3D shape instantiation, facilitating enhanced surgical guidance. Additionally, the current surgical trend is shifting towards intraluminal procedures and endoscopic surgery, driven by the emphasis on early detection and intervention. To guide endoscopes accurately during procedures, navigation techniques have been developed, utilizing learning-based depth estimation, visual odometry, and simultaneous localization and mapping (SLAM) with endoscopic images. Depth estimation from endoscopic images is crucial for camera motion estimation and 3D environment mapping. This process is challenged by the scarcity of high-quality training data and the textureless nature of surgical scenes. Various approaches have been proposed to address these challenges, including self-supervised depth estimation, domain transfer learning, and image-to-image translation methods [44]. Visual odometry estimates camera pose using consecutive video frames, with CNN-based approaches adopted for feature extraction and dynamics estimation. However, the feasibility of these approaches has mainly been validated in lung and gastrointestinal phantom data. Real-time 3D reconstruction and localization are vital for navigation in dynamic tissue environments. Traditional SLAM algorithms face limitations in surgical settings due to the deformable nature of tissues [45]. Researchers have proposed novel approaches such as compensating for tissue motion caused by respiration, using monocular SLAM in hernia repair surgery, and implementing dense deformable SLAM for stereoendoscope localization. In endovascular interventions, intravascular ultrasound (IVUS) has emerged as a valuable tool for intraoperative guidance. Researchers have developed frameworks for 3D vasculature reconstruction using IVUS and electromagnetic sensing data fusion [32]. Improved methods have been proposed to handle errors and uncertainties, enhancing the efficiency of data fusion, and reducing the need for preregistration between preoperative CT data and electromagnetic sensing data [32].

Learning methods have also been employed for tracking soft tissue in MIS. Mountney P and Yang G [46] developed an online learning framework that continuously updates the feature tracker by selecting suitable features using decision tree classification. Ye M et al. [47] introduced a detection method that integrates a structured support vector machine (SVM) with an online random forest to retarget a predefined optical biopsy region on the gastrointestinal (GI) tract’s soft tissue surfaces. Wang R et al. [48] employed a statistical appearance model to differentiate the organ from the background in their region-based 3D tracking algorithm. Their validation results demonstrated that the application of learning techniques can improve the stability of tissue tracking in terms of deformations and illumination changes. On the other hand, augmented reality enhances surgeons’ intraoperative vision by superimposing preoperative images semi-transparently onto the area of interest [49]. Wang J et al. [50] employed a projector to display the AR overlay for oral and maxillofacial surgery. They utilized a 3D contour matching technique to calculate the transformation between the virtual image and real teeth. Pratt P et al. utilized the Hololens, a head-mounted AR device, to project a 3D vascular model onto patients’ lower limbs [51]. Given the challenge of projecting overlays onto markerless deformable organs, Zhang X et al. [52] introduced an automatic registration framework for AR navigation, employing iterative closest point and RANSAC algorithms for 3D deformable tissue reconstruction. In conclusion, developing computer-assisted guidance based on visual observations necessitates enhancing localization and mapping performance in challenging conditions such as textureless surfaces, variable illumination, and restricted fields of view. Another significant challenge is the deformation of organs/tissues during surgery, creating a dynamic and uncertain environment despite thorough preoperative planning. While AI technologies have made strides in detection, segmentation, tracking, and classification, further research is needed to extend these capabilities to more complex 3D applications. Moreover, in surgery, AI algorithms must efficiently assist surgeons in real-time, particularly in the development of Augmented Reality or Virtual Reality systems where frequent interactions occur between surgeons and autonomous guidance systems, or during remote surgery involving MTBs in disparate locations. Besides visual data, future AI technologies must integrate multimodal data from diverse sensors to achieve more precise perception of complex environments. Additionally, the increasing utilization of micro- and nano-robotics in surgery will introduce new challenges in guidance.

## 3. Medical Oncologist’s Perspective

**Figure 2:**
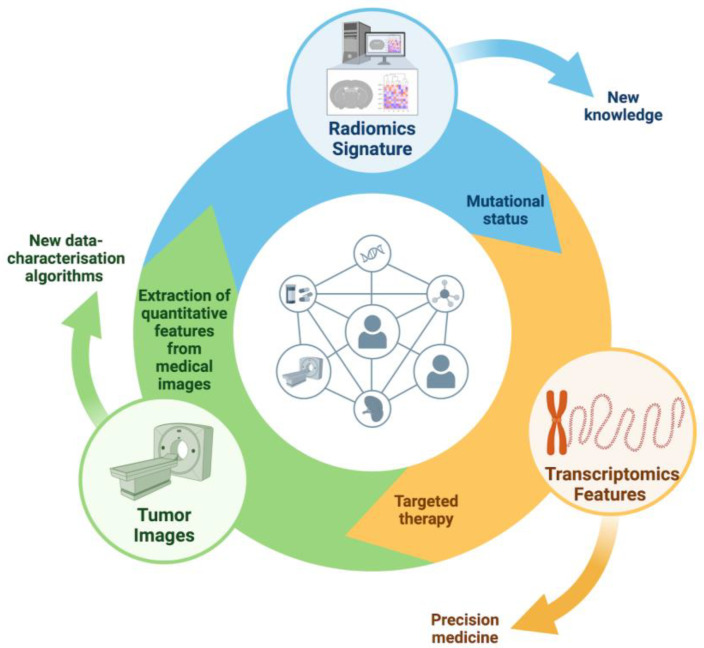
Integration of AI in Precision Oncology. AI algorithms analyze tumor images to extract quantitative features, forming a radiomics signature. This signature, combined with transcriptomics data, identifies mutational status and informs targeted therapies. This feedback loop enhances precision medicine by continuously improving data-characterization algorithms and treatment strategies.

The integration of AI in oncology, particularly from the viewpoint of medical oncologists, is revolutionizing cancer care. AI applications encompass molecular profiling, treatment selection, predictive modeling for drug response, and clinical trial design. In molecular profiling, AI algorithms analyze multi-OMICs data to enhance molecular characterization, tumor grading, and clinical decision-making [53]. Radiogenomics, an emerging paradigm, combines imaging-derived parameters with genomic data to provide insights into tumor biology [54]. Radiomics, leveraging quantitative features from medical images, aids in predicting treatment response and patient outcomes [55,56]. Moreover, AI-driven platforms optimize clinical trial protocols and enhance patient recruitment by matching individuals with suitable trials based on their molecular profiles [57,58]. Despite challenges, AI holds immense promise in transforming oncology, laying the foundation for more personalized and effective cancer treatments. The different applications of AI in medical oncology are summarized in Table 2 and Figure 2.

### 3.1. AI Applications in Molecular Profiling and Treatment Selection

In Oncology, AI algorithms could be applied in imaging and pathology unraveling the molecular mechanisms via integrative analysis of multi-OMICs data and could significantly improve the prediction accuracy for molecular characterization, grading of tumors, clinical decision-making, and prognosis. In the last decade, the new idea that diagnostic images contain more data than human eyes can see has emerged, and the extraction and analysis of radiomic features may allow obtaining biological data in a non-invasive way. Radiogenomics is a relatively new paradigm, used for the integration of imaging-derived parameters and genomic data of the tumor. The prognostic and predictive value of radiomics in colorectal cancer had been well studied, with several studies demonstrating the possibility to correlate radiomic features to *RAS* status, improving patient selection for cancer therapy and predicting response to treatment.

Indeed, mutations in *RAS* were strongly associated with worse overall survival and these genetic alterations are a predictive biomarker of resistance to anti-EGFR therapy in metastatic colorectal cancer (mCRC). Texture parameters derived by MRI from liver metastases of colon cancer patients could allow stratifying the patients according to *RAS* mutation status [53]. Similar results have been demonstrated in primary colon cancers, showing that the CT based-radiomics signature was significantly associated with *KRAS/NRAS/BRAF* mutations and this approach may be useful for analysis of tumor genotype [54]. The assessment of the molecular profile is crucial step to guide treatment decisions in patients affected by advanced lung cancer. Current data supporting that radiogenomic approach can identify epidermal growth factor receptor (EGFR) expression [59] or be utilized to help to differentiate between adenocarcinoma and squamous cell carcinoma of the lung [60] predicting diagnosis, prognosis, and optimal therapy. The relationship between radiomic data and gene expression has also provided great insight in the prostate cancer (PCa) risk stratification holding a promising future in the growing era of personalized medicine. PCa with poor prognosis seems to have genomic alterations [61], such as due to *PTEN* loss. Retrospective analyzes investigating the associations between the MRI imaging features and the *PTEN* expression of PCa showed a correlation between Gleason score and *PTEN* expression [62]. Using the cell cycle progression score and MRI data, radiogenomics analysis is able to predict Gleason scores in the tumor suggesting that the management of the early stages PCa could benefit, by performing MRI-targeted biopsy coupled with molecular profile [63]. In an analysis including clinical, imaging, and genomic datasets for PCa patients, four biomarkers were highly correlated with aggressiveness on radiomics features, proposing a model that could improve the prediction accuracy for disease stage and the characterization of PCa aggressiveness [64]. Promising results have revealed that radiomic signatures can be a non-invasive tool to distinguish molecular subtypes among triple negative breast cancer (TNBC) [65]. Furthermore, these findings describe the prognostic role of radiomic features capturing peritumoral heterogeneity and allow the prediction of recurrence-free survival and overall survival. 

Among wide spectrum of AI–based applications, digital pathology is emerging as novel analytical strategies for realizing new information derived from standard histology to guide treatment selection and biomarker development. With the advent of digital histology, DL can be used to pinpoint more minute details identifying a great amount of information from traditional histology to discover pathogenic mutations, gene expression patterns, clinical biomarkers and survival outcomes [66]. In this context, a recent study introduces a novel approach for identifying patients with PCa with higher risk for early recurrence after prostatectomy; an AI-powered platform extracted visual and subvisual morphologic features able to identify driver regions predictive of recurrence of PCa after prostatectomy [67].

### 3.2. Predictive Modeling for Drug Response and Personalized Therapy

Radiomics involving a large number of quantitative features from medical images could represent a promise to assess the response of tumors to various treatments, helping clinicians understand how well a patient is responding to therapy or to contribute to the development of personalized treatment. In their evaluation of the potential role of radiomic signatures to predict response to irinotecan, 5-fluorouracil, and leucovorin (FOLFIRI) alone or in conjunction with an anti-EGFR agent, Dercle L et al. found that in the group receiving cetuximab, radiomic signatures outperformed current biomarkers (*KRAS* status) for the detection of treatment sensitivity [55]. The development of a signature based on routinely acquired CT scans to guide the clinical decision to continue EGFR targeted therapies could be an innovative tool to be widely incorporated into clinical practice at minimal cost to detect EGFR-resistant tumors. Regarding to liver metastases in mCRC patients, a retrospective analysis of TRIBE2 trial showed that radiomic features evaluated at pre-surgery CT scan were associated with higher risk of relapse or death after surgery in the subgroup of patients undergoing liver metastases resection with radical intent [68]. Based on these published findings, the radiomics-approach might ideally be objective measures of tumor aggressiveness and biology impacting in the clinical decision-making process helping clinicians to identify patients who may benefit from surgery or other locoregional techniques. Recent studies have shown radiomics of NSCLC can help to evaluate treatment efficacy and predict treatment-related outcomes [69]. Available data showed that the change in radiomic measures between baseline and post-treatment CT images affected the sensitivity to immune checkpoint inhibitors (ICIs) in advanced NSCLC patients [70]. Personalized decision making for patients with advanced NSCLC may benefit from the integration of radiomic features for selecting candidates who will have the greatest benefit from immunotherapy while avoiding others of adverse side effects. Using only MRI exams acquired at the pre-treatment baseline, radiomics was predictive of recurrence-free survival for breast cancer patients undergoing neoadjuvant chemotherapy [56]. The combination of clinical, histological, and genetic data with quantitative radiomics characteristics may make it easier for physicians to develop patient-tailored treatment in the era of personalized medicine.

Regarding digital pathology and prediction, a proof-of-concept study investigated a potential predictive role of a tumor microenvironment driven by AI in advanced NSCLC patients receiving ICIs. In this analysis, AI was able to distinguish between three immune phenotypes (inflammatory, immunological-excluded, and immune-desert) associated with survival and response to ICI in two different cohorts with advanced NSCLC patients suggesting its potential predictive role [71].

### 3.3. Integrating AI in Clinical Trial Design and Patient Recruitment

Oncology is currently the widest field of application of AI in medical research, with 50% of published papers devoted to the use of AI between 2017 and 2021 matching cancer-related fields [72]. As per May 2021, 71 AI-based devices received official clearance by the U.S. Food and Drug Administration (FDA) for clinical use, mostly addressing tumor radiology (55%) and pathology (20%). Although breast cancer represents the largest area of application (31%), followed by lung (8%), prostate (8%) and colorectal cancers (7%), the largest amount of AI-based devices (34%) has been designed for a wide spectrum of solid organ malignancies, spreading hope for an agnostic-based approach of AI resources that may address the vastly unmet need of care for rare malignancies [73]. More than 80% of the currently AI-based approved devices cover the field of cancer diagnostics. However, because most AI technologies with a potential clinical impact have been developed on the basis of radiology and pathology imaging, emerging tools may foster AI application to conduct clinical trials, including design, and recruitment [73,74].

Indeed, clinical trial optimization is a crucial issue in medical research. According to Wong C et al., the success rate of drug-development programs is only 12%, suggesting that there is significant room for improvement for the administration of clinical trials [57]. Reasons for failure are the lack of efficacy or safety of the experimental drug, slow accrual, and participant drop-out. AI can support researchers in each phase of drug development. As an example, natural language processing (NLP) software (i.e., Trials.ai) can collect and analyze written words of publicly available data, including peer-reviewed journals, drug labels, and clinical trial datasets (https://www.trials.ai/). Because the computational speed of AI largely outweighs that of human researchers, while collecting data from a greater source, AI has the potential to improve the accuracy of protocol design by optimizing thresholds of outcome measures (i.e., study endpoints) and cohort sample sizes, which are currently established based on the researchers’ scientific background, the estimated sensitivity of specificity of the study, and its pragmatic financial sustainability [75]. AI has also proved accurate at predicting Progression-Free Survival (PFS) and Overall Survival (OS) in 939 patients enrolled in randomized clinical trials with metastatic or locally advanced colorectal adenocarcinoma, pancreatic adenocarcinoma, melanoma and lung cancer and molecular, transcriptomic, and proteomic data available enrolled in randomized clinical trials [76]. These data suggest that AI software may reshape clinical trial design replacing a control/placebo arm with a virtual arm consisting of synthetic data, alleviating cohort dimensions, study duration, costs and logistics in recruiting sites, while minimizing the risk of exposing patients to an ineffective and toxic drug following the standard random-allocation procedure [72]. Causal AI can even simulate in silico trials, where both control and efficacy arms are grounded synthetic data [77], and assess the likelihood of success in phase transitions [78], with obvious advantages for pharmaceutical industries to tailor their research efforts in human research. Patient recruitment is another potential field of application of AI, underlined by the low accrual rate (3–5%) of cancer patients in clinical trials, as compared to roughly 20% who may actually be eligible [79]. The main reasons for poor recruitment are protocol complexity, lack of awareness of the trial, emotional fear of participation, and lack of interest to participate. The growing amount of -omics data have further tangled this experimental scenario, with inclusion and exclusion criteria matching specific genomic, transcriptomic, proteomic or metabolomic profiles but AI technology can overcome such overwhelming complexity [72]. Indeed, a pivotal study conducted in the Mayo Clinic showed that the NPL software Watson for Clinical Trial Matching (CTM) cognitive system increased the accrual of breast cancer patients for systemic therapy trials from 3.5 to 6.3 patients/months over 18 months following the software implementation [58,78]. 

Taken together, these data suggest that AI has a striking potential to transform research in Oncology, but several unsolved challenges still remain. Indeed, large-scale AI validation for research purpose is still limited by not standardized reporting of clinical information, which dampens the integration of differential sources of data, the uneven spread of -omics data, tackling their reproducibility and building of an accurate omics-based AI software, and the lack of a proper regulatory framework for protected health-data repositories [72].

While AI offers significant advancements in oncology, its integration into clinical practice presents several challenges that must be thoughtfully addressed. The implementation of AI demands that medical oncologists acquire new skills, particularly in interpreting and critically assessing AI-derived data. Ongoing education and close collaboration with AI experts are crucial to ensure that AI technologies are effectively applied in patient care. Additionally, it is important to consider the ethical dimensions of AI-driven decisions, especially in cases where there may be substantial risks or where AI recommendations differ from traditional clinical judgments.

Moreover, disparities in the availability of AI technology pose a considerable obstacle. The access to advanced AI tools can vary widely between different healthcare institutions and regions, potentially leading to unequal standards of care. It is vital to ensure that AI applications are made accessible and useful across all healthcare environments to promote fair and widespread adoption. Overall, while AI holds remarkable potential to revolutionize cancer treatment, its successful integration into oncology will depend on a balanced approach that includes technological innovation, strong clinical expertise, ethical reflection, and a commitment to ongoing education and interprofessional collaboration.

## 4. Radiation Oncologist Perspectives

**Figure 3:**
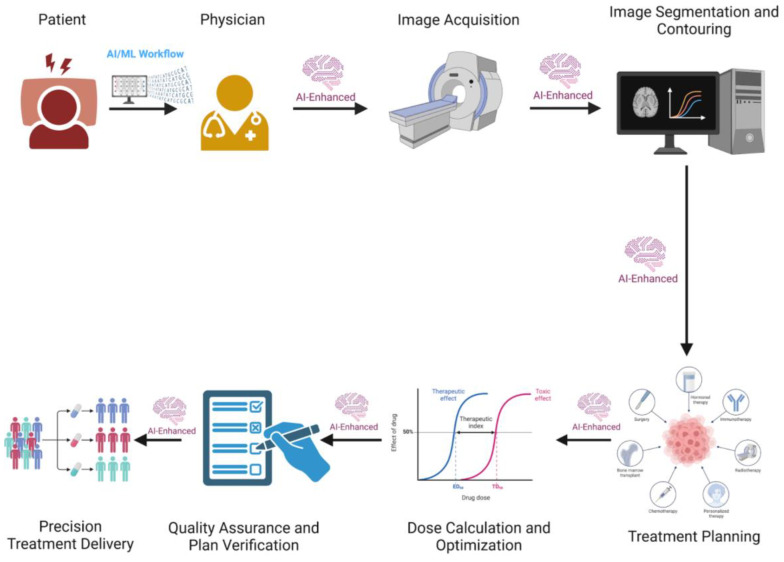
AI-Enhanced Workflow for Multidisciplinary Tumor Board (MTB) in Radiotherapy. The workflow includes patient consultation, physician assessment, image acquisition, image segmentation and contouring, treatment planning, dose calculation and optimization, quality assurance and plan verification, and precision treatment delivery. Each stage is augmented by AI to improve accuracy, efficiency, and patient outcomes.

The application of AI in radiation oncology has become increasingly prominent, offering transformative potential across various facets of radiation oncology (RO). In this discourse, we will describe three pivotal aspects where AI intersects with RO, elucidating its profound implications and promising advancements. Firstly, we will delve into the utilization of AI in contouring, treatment planning, optimization, and adaptive workflow, demonstrating how AI contributes to decision support systems, data mining, and advanced imaging analysis. This will streamline treatment workflows and enhance clinical outcomes. Secondly, we will explore the application of AI in patient selection, outcome prediction, and side effect anticipation, highlighting its role in adaptive radiation therapy and real-time decision support. This showcases AI’s ability to aid MTBs in personalized patient management by swiftly analyzing complex medical data. Lastly, we will address uncertainties and limitations inherent in AI for radiation oncology, emphasizing the importance of standardized protocols, extensive clinical trials, and regulatory measures to harness the full potential of AI while ensuring patient safety and efficacy in oncological radiotherapy. Through this exploration, we aim to illuminate the transformative power of AI in revolutionizing radiation oncology practice, ultimately benefiting cancer patients worldwide. The different applications of AI in radiation oncology are summarized in Figure 3 and Table 3.

### 4.1. AI-Enhanced Radiotherapy Workflow (Contouring, Treatment Planning, Adaptive and Advanced Imaging Analysis)

In the last ten years, AI has played a significant role in addressing medical challenges, including cancer. DL, a subset of AI, stands out for its ability to automatically extract features and process vast amounts of complex data efficiently. With the help of extensive medical data and advanced computational tools, AI, particularly DL, has been utilized in various areas of oncology research to improve cancer diagnosis and treatment. These applications span from early cancer detection, diagnosis, classification, and grading, to molecular profiling of tumors, forecasting patient outcomes and treatment responses, customized treatment plans, streamlining radiotherapy processes, innovation in anti-cancer drug discovery, and conducting clinical trials [80]. AI is pivotal in developing DSS, and its application in healthcare is growing rapidly [77]. In this context, ARCHERY is a prospective, non-randomized study aimed at evaluating the quality and economic implications of AI-based automated radiotherapy treatment planning for cervical, head and neck, and prostate cancers. These cancers are prevalent in LMICs and rely on radiotherapy as the primary curative treatment modality. By 2030, it is projected that the number of new cancer cases globally will increase to 21 million; 3 million annually, with approximately 70% of these cases occurring in LMICs. Radiotherapy (RT) plays a crucial role in the management and cure of several common cancers in these regions, such as cervical, prostate, and head and neck cancers, with 50% of these patients requiring RT at some stage of their disease. However, access to RT is limited, with only 10% of patients in low-income countries and 40% in middle-income countries having access to this treatment. Resource constraints have led to long waiting times for treatment, resulting in cancer progression, increased morbidity, and poorer survival outcomes. The pre-treatment RT workflow can typically take up to four weeks in high-income countries and up to twelve weeks in LMICs due to patient demand and workforce shortages. The WHO has set a target for RT to be available to 80% of the global population by 2025, acknowledging the primary obstacle to achieving this goal is a critical shortage of the specialized workforce necessary to deliver RT. The increasing demand for RT necessitates a scalable solution to address these challenges. In this regard, a recent WHO report underscored the potential of digital technologies, such as AI, to advance universal health coverage and achieve Sustainable Development Goals, including ensuring equitable access to and affordability of treatments. The Radiation Planning Assistant (RPA) is an AI-based software designed to automate two crucial components of the RT planning pathway: (1) contouring anatomical areas at risk of tumor spread (CTVs) and those at risk of radiation damage (OARs), and (2) defining the position, size, and shape of the radiation beams targeting the organs. The AI-based contouring models included in the RPA were developed by the University of Texas MD Anderson Cancer Centre, which also created the user interfaces, AI-based planning for conformal RT treatments, integrated quality assurance tools, and the training and testing of the knowledge-based planning component of the automated planning software, a function of the Eclipse treatment planning system (Varian Medical Systems). To our knowledge, the RPA is the only application ready for clinical use in head and neck, cervical, and prostate cancers that can both contour CTVs and OARs and generate an optimized treatment plan [78]. AI in RO significantly impacts clinical decision support, data mining, and advanced image analysis, automating repetitive tasks, optimizing time, and modelling patient and clinician behavior in varied contexts. The implementation of AI and automation in RO and IRT can effectively facilitate all steps of the treatment workflow, including patient consultation, target volume delineation, treatment planning, and treatment delivery. AI can enhance clinical outcomes through predictive models and DSS optimization, reducing time-consuming repetitive tasks, lowering healthcare costs, and improving treatment quality assurance and patient support in IRT. RT is utilized in 45–55% of newly diagnosed cancers and even in advanced disease. However, RT demands high standards of training and quality assurance due to its technologically complex and advanced nature. The adoption of AI methods can significantly improve treatment quality and overall effectiveness [77]. In radiation oncology, the AI revolution has also provided automated support for various parts of the clinical radiotherapy workflow: target and tissue segmentation, treatment planning, radiotherapy delivery, and treatment response assessment. Radiotherapy treatment planning, especially inverse treatment planning, is a labor-intensive process that can take hours or even days to complete. Future treatment planning using a robust AI agent can be efficient and effective with minimal human intervention [79]. AI offers the opportunity to automate radiotherapy (RT) planning, minimizing variability in patient treatment. DL techniques can be used to automate contouring and generate RT plans using clustering analysis or Pareto-guided navigation after chemotherapy and image-guided radiotherapy. AI can automate contouring of target volumes and organs at risk (OAR) across different imaging modalities and tumor locations, as well as help adapt RT plans during treatment. Image quality can be improved through AI-based denoising techniques for low-dose CT images, optimizing signal-to-noise ratios while maintaining low patient doses. AI technologies have also shown promise in reducing acquisition times for MRI scans and reducing radiation doses for CT and PET scans. AI models can compensate for data imbalances during training by generating synthetic data that follows real-world data distributions. These technologies have the potential to improve toxicity prediction, automate RT planning and optimization, select patients for clinical trials, and reduce clinical workload in radiation oncology. Education and training resources are critical for radiation oncology staff to understand data sourcing, curation, ethics, and interpretation of AI technologies. Patient involvement in the development and implementation of AI technologies is essential to ensure transparency and trust in these tools. Standardized reporting and evaluation of AI technologies is necessary for clinical integration and widespread adoption. Despite challenges such as lack of generalizability and limited validation studies, AI holds promise for transforming radiotherapy practice. Through standardized reporting, integration with existing healthcare systems, and collaboration between providers, AI technologies can improve the accuracy, efficiency, and quality of radiotherapy treatment [80].

### 4.2. Artificial Intelligence in Prediction of Radiotherapy Outcomes and Toxicity

AI and ML are being integrated into radiation oncology to improve the prediction of radiation therapy outcomes and toxicity. AI has the potential to transform radiation oncology by improving the accuracy, efficiency, and quality of radiation therapy through its ability to recognize complex patterns in medical data [81]. Radiomics is a rapidly developing area of research in this field. It involves extracting quantitative metrics, known as radiomics features, from medical images. These features include information on tissue and lesion characteristics, such as heterogeneity and shape. They can be used independently or with demographic, histological, genomic, or proteomic data to answer specific clinical questions [82]. Integrating this data can provide a deeper understanding of cancer characteristics. Radiomics aims to improve decision-making in precision medicine by studying the correlations between these characteristics and patient prognosis [83,84]. Radiomics can provide useful information about the effectiveness of a treatment by analyzing changes in predefined features extracted from diagnostic images over time [85]. ML-based models have shown high accuracy in predicting radiotherapy-induced side effects. However, their clinical implementation is hindered by challenges such as low interpretability [85,86]. For instance, artificial intelligence can extract radiomic features from 3D dose maps, which have been proven to enhance the prediction of acute and late lung toxicities in lung cancer radiotherapy, as opposed to traditional models based on clinical factors and dose volume histograms [87]. Genetically-based risk models are being developed to predict radiotherapy toxicity. These models incorporate single nucleotide polymorphisms and could improve personalized treatment planning [88]. In summary, AI and ML are valuable tools in radiation oncology. They can predict treatment outcomes and toxicity by analyzing complex medical data. These technologies contribute to more accurate and personalized radiotherapy. Predictive models can be improved by integrating radiomic features, genetic factors, and AI-based imaging analyzes into them. This improvement promises to enhance clinical decision-making and patient care. However, to enable broader clinical adoption, we must address challenges such as interpretability, validation, and standardization.

### 4.3. Addressing Uncertainties and Limitations in AI for Radiation Oncology

AI plans to transform different fields, including radiation oncology [89]. Despite the numerous articles published in recent years, there are no standardized protocols available to evaluate the efficacy of the developed tools. This hinders the clinical assessment of the proposed AI methods [90]. Proposed as a way to improve the quality, uniformity, and effectiveness of multiple stages in delivering radiation, AI shows potential for tasks including self-segmentation and automated treatment planning. Nevertheless, worries have arisen about the possible loss of domain expertise between doctors and physicists because of greater automation. Proficiency heavily depends on practical experience; however, automation impedes opportunities to acquire first-hand experience in creating treatment plans or segmentations within the clinical workflow. Models must adjust over time due to the ever-changing nature of clinical workflows, fractionation patterns, and medical devices. Regular updates with new information are necessary to show changes in medical practices, highlighting the importance of having particular knowledge to produce this updated data [91]. Thanks to its automated procedures, TPS has efficiently decreased the time needed for plan generation. Nevertheless, despite the boosted efficiency and consistency, TPS’s current techniques have not impacted the quality of the plans. Research has shown that plans created by machines are compliant with clinical standards. Nevertheless, some results emphasize the importance of human intervention or modification in the ATP process to ensure a sufficient level of quality and safety. A major limitation in using DL for oncological radiotherapy is the small number of available datasets [92]. This issue occurs because radiotherapy often involves only small datasets. As a result, choosing the appropriate algorithm for a particular situation can lead to changes of up to 32% in the expected results. To address these limitations, it is vital to conduct extensive clinical trials to assess the effectiveness of AI in the field of oncological radiotherapy. Additionally, it is essential to establish norms and principles for the smooth integration of AI technology in clinical settings. The realization of AI advantages and the assurance of safeguarding patients can solely be accomplished through a sturdy basis of scientific proof and apt regulatory measures.

In synopsis, despite the encouraging prospects AI offers for improving oncological radiotherapy, it is crucial to confront current uncertainties and constraints. Merely by doing so can we obtain the full potential of this technology and endow cancer patients with safe and effective treatments.

## 5. MTBs Perspective

Integrating AI into the MTB can greatly enhance the efficiency and effectiveness of cancer care. AI systems can analyze large datasets, including genomic information, imaging results, and clinical records, to identify patterns and correlations that may not be immediately apparent to human clinicians. This capability allows AI to provide valuable insights into tumor characteristics, potential treatment responses, and patient prognoses. Cancer treatment options are becoming more varied, and guidelines are changing rapidly. As a result, medical personnel must have access to comprehensive medical information, necessitating considerable time spent updating their knowledge alongside providing direct patient care. For instance, oncologists work an average of 56.7 h per week, spending approximately 4.6 h updating and maintaining their knowledge in the field. As cancer treatment methods become more diverse and complex, it is crucial to have objective evidence and opinions from medical professionals in various fields to determine the most appropriate treatments for individual patients. Since the 1980s, MDTs have been used to improve the quality of care for cancer patients. These teams, consisting of medical professionals from different specialties, collaborate to define treatment plans for each patient. MDTs improve patient outcomes and reduce the workload of medical staff but often lack timely medical updates. To address this challenge, CDSS have been developed [6]. A CDSS is an AI-based application designed to reduce the time medical staff spend evaluating evidence-based practices. This approach involves using multifactorial decision support systems (DSSs) and continuously learning AI platforms that integrate all available data—clinical, imaging, biologic, genetic, and cost-related—to produce validated predictive models [7]. Moreover, AI can streamline administrative and logistical aspects of the MTB. For instance, AI-driven tools can automate the collection and organization of patient data, ensuring all relevant information is readily available for board members. This reduces the time spent on manual data entry and allows clinicians to focus on critical case discussions. IBM has developed software designed to analyze the meaning and context of both structured and unstructured data in clinical notes and reports, efficiently assimilating key patient information written in plain English. By integrating attributes from the patient’s file with clinical expertise from Memorial Sloan Kettering, along with external research and data, Watson for Oncology identifies and ranks potential treatment plans and options. This software, formerly known as AI CDSS Watson for Oncology (WFO), has been tested in several contexts within the MTBs [93,94,95] and is now equipped with advanced generative AI capabilities powered by foundation models and traditional ML in a comprehensive studio spanning the AI lifecycle. Generally, decisions made by WFO showed a high concordance rate with MTBs, and applying WFO is likely to facilitate a multidisciplinary team approach, also saving time for easier cases. Several similar approaches have been developed, using other self-developed smart virtual assistants capable of understanding different free-text reports to facilitate and standardize the language and interpretation of MTB discussions [95,96,97,98,99]. Other approaches have used ChatGPT, an AI chatbot that employs natural language processing to create human-like conversational dialogue, in the context of MTBs. However, the current version cannot provide specific recommendations, emphasizing the need for dedicated AI software for this purpose [100,101,102]. Finally, Kasprzak J et al. trained a ML model on clinical data to determine whether molecular profiling should be performed for a patient discussed within an MTB, optimizing the time of this decision which can significantly impact outcomes [103].

In summary, the inclusion of AI in the MTB can enhance diagnostic precision, personalize treatment plans, predict patient outcomes, and streamline administrative tasks. By integrating AI, the MTB can provide more comprehensive, timely, and effective cancer care, ultimately improving patient outcomes and advancing the field of oncology. The different applications of AI in MTB are summarized in Table 4.

## 6. Discussion

AI is emerging as a powerful resource to improve the analysis and management of medical conditions, including cancer. AI has many applications, including medical imaging and pathology. Integrating AI into clinical workflows can improve diagnostic accuracy and personalize treatments [104]. This study revealed some key points, such as:

- AI-based tools are already influencing surgical planning and predicting complications, recurrences, and therapeutic responses in medical imaging. This is advancing towards personalized medicine;
- AI’s ability to analyze big data can help discover new biomarkers and improve cancer screening, diagnosis, treatment, and prognosis. This can lead to better clinical outcomes [105];
- The use of deep learning-based AI in cancer pathology can enhance diagnostic accuracy, reduce the workload of pathologists, and support high-level decisions. Despite the challenges of algorithm validation and interpretation, this technology has the potential to revolutionize cancer diagnosis [106].

In addition to imaging and medical pathology, AI has also found application in other areas of healthcare, such as genomics and drug discovery. By exploiting AI algorithms, researchers and doctors can analyze large amounts of genomic data to identify genetic markers associated with certain diseases, including cancer. This information can be used to develop targeted therapies and personalized treatment plans for patients. Furthermore, CDSS powered by artificial intelligence can help healthcare professionals make more informed decisions by providing evidence-based recommendations and warnings. These systems can analyze patient data, medical literature and clinical guidelines to offer real-time guidance on diagnosis, treatment options and medication management. By integrating artificial intelligence into clinical workflows, healthcare professionals can improve the accuracy and efficiency of their decision-making processes, ultimately leading to improved patient outcomes. Indeed, the introduction of digital cancer board solutions has significantly reduced case discussion time, standardized the case presentation process and potentially increased efficiency in therapeutic decision-making. Collaboration between doctors and artificial intelligence for decision-making aims to achieve team performance that exceeds the performance of either humans or artificial intelligence alone. The advice of an AI assistant can help users make decisions, but factors such as the experience of the user base and the complementary fine-tuning of humans and AI have a significant impact on the overall performance of the team [6]. AI systems need to be trained to focus on the needs of medical staff to optimize team performance. Research suggests that integrating AI in a way that complements human expertise, building mutual trust, and optimizing AI algorithms for teamwork rather than individual accuracy can accelerate decision-making in multidisciplinary teams. It is crucial to understand team dynamics and effectively manage human-AI interactions to take full advantage of AI in team decision-making. 

However, the integration of AI into oncology varies significantly across medical specialties, each facing distinct challenges and opportunities. For surgeons, AI holds promise in enhancing decision-making and providing intraoperative guidance. They are particularly interested in AI applications that improve the precision and safety of procedures, such as real-time anatomical recognition and decision support systems. However, concerns remain about the reliability of AI in complex, high-stakes environments where human judgment is crucial [15]. Medical oncologists view AI as a powerful tool for molecular profiling and personalized treatment planning. The ability of AI to analyze large datasets, such as genomic information, and predict treatment responses is a significant advantage. However, integrating AI into routine practice poses challenges, particularly in interpreting AI-generated data and ensuring that AI tools are user-friendly and seamlessly incorporated into existing workflows [72]. Radiation oncologists increasingly adopt AI for treatment planning and optimization. AI’s ability to enhance contouring accuracy and predict treatment outcomes is highly valued in this specialty. However, concerns about data quality, the need for continuous model updates, and the potential reduction in hands-on experience for developing expertise in treatment planning are significant barriers to adoption [84]. 

The integration of AI into clinical practice across these specialties is not without its challenges. A key issue is the need for specialized training to ensure effective use of AI tools, especially in surgery, where errors can have immediate, life-threatening consequences [49,107]. In medical oncology, the challenge lies in interpreting complex AI-generated data and ensuring that AI simplifies, rather than complicates, decision-making. For radiation oncologists, maintaining the quality and consistency of AI tools amid rapid technological advancements is a primary concern. Despite these challenges, AI offers substantial opportunities. It has the potential to enhance the precision of cancer treatment, improve patient outcomes, and streamline clinical workflows. Surgeons can benefit from real-time decision support during complex procedures, potentially reducing complications. Medical oncologists can employ AI to identify the most effective treatments based on a patient’s molecular profile, paving the way for more personalized care. Radiation oncologists can optimize treatment plans to ensure patients receive the most accurate and effective radiation doses. Overall, AI also shows promise in enhancing the collaborative nature of MTBs [95,98,99]. By providing comprehensive data analysis and predictive modeling, AI can facilitate more informed decision-making across disciplines, improving the efficiency and effectiveness of MTBs and leading to better patient outcomes. However, it is crucial that AI tools complement, rather than replace, the expertise of human clinicians. The successful integration of AI into MTBs will depend on fostering mutual trust between AI systems and healthcare professionals, ensuring that AI enhances, rather than undermines, interdisciplinary collaboration.

Recently, Grunhut J referred to AI as the “elephant in the MTB room”, as these technologies are transforming the landscape of oncology and introducing new ethical complexities [108]. AI technologies raise significant ethical concerns, particularly regarding the potential to perpetuate biases found in their training data [109,110,111]. For instance, Sap M et al. [112] demonstrated that algorithmic systems, especially in content recommendation, can inadvertently reinforce stereotypes, leading to reduced diversity of accessible information and the creation of echo chambers [113]. Addressing these biases requires proactive measures such as continuous monitoring, regular audits, and transparency in the development and deployment processes [114,115]. Interdisciplinary collaboration among AI developers, policymakers, and ethicists is essential to ensure that AI systems are equitable and unbiased [116]. Establishing guidelines and regulations that focus on fairness, accountability, and transparency is critical to the responsible development and use of AI [117,118]. Despite the early stage of AI regulation [119], there is a growing call for tailored regulations to address the unique ethical challenges posed by AI [120,121]. Therefore, robust legal and ethical frameworks are necessary to guide responsible AI practices. Additionally, privacy and confidentiality are also crucial when handling sensitive medical data within AI systems, which must comply with existing or forthcoming privacy regulations. Ensuring secure management of patient information involves robust encryption, access control mechanisms, and data anonymization techniques, especially when integrating data from various sources. Standardized interfaces and protocols are recommended to ensure secure data transmission [122]. Compliance with GDPR and ISO 27001 standards [123] is essential to align with industry best practices [124]. As data-sharing models evolve, some datasets may become publicly available, while others may be restricted to consortia or healthcare systems, potentially with access fees. The field of cybersecurity continues to advance, addressing challenges such as stabilizing image reconstruction neural networks [125]. Incorporating robust security measures and adhering to relevant regulations can lead to a secure AI platform capable of integrating and analyzing medical data from diverse sources.

## 7. Conclusions

The integration of AI in MTBs holds significant promise for enhancing cancer care. AI technologies, including machine learning and deep learning, offer the potential to improve diagnostic accuracy, personalize treatment plans, and predict patient outcomes more effectively. These advancements facilitate more informed decision-making, optimizing the efficiency and effectiveness of cancer treatment. Despite these benefits, the implementation of AI in oncology is not without challenges. The variability in anatomical structures, the need for standardized protocols, the small size of training datasets, and the necessity for regular model updates present significant hurdles. Human intervention remains essential to ensure quality and safety in AI applications, particularly in clinical settings. Overall, AI’s integration into MTBs can lead to more comprehensive, timely, and effective cancer care. By enhancing the capabilities of MTBs, AI can improve patient outcomes and advance the field of oncology. Continued research and development, alongside rigorous validation and standardization efforts, are crucial to fully realizing the potential of AI in cancer treatment.

## Figures and Tables

**Table 1:** AI Applications in Surgical Performance, Preoperative Planning, and Intraoperative Support.

Category	Details
AI to Enhance Surgical Performance and Education	High rates of preventable adverse events in surgery highlight the need for improved judgment and decision-making. AI and machine learning, particularly computer vision, are used to develop algorithms for error reduction and real-time guidance. Challenges include variability in anatomical structures and expert cognitive behaviors. Tools like GoNoGoNet and CholeNet offer real-time guidance by identifying safe and hazardous areas during surgeries like laparoscopic cholecystectomy. DeepCVS model predicts critical views of safety (CVS) in surgical procedures.
AI to Enhance Preoperative Setting	AI aids in surgical planning using medical records and imaging (X-ray, CT, MRI). Techniques include anatomical classification, detection, segmentation, and registration. Deep learning enhances these tasks but faces challenges like generalizability and explainability. Collaborative efforts and personalized data integration are essential for early detection and treatment.
AI for Intraoperative Support	AI in MIS provides improved visualization and localization through shape instantiation, endoscopic navigation, tissue tracking, and augmented reality. Advances in 3D reconstruction from 2D images and navigation techniques like SLAM help guide endoscopes. Tissue tracking is improved with learning-based methods. Augmented reality enhances intraoperative vision by overlaying preoperative images. Challenges include textureless surfaces, variable illumination, and organ deformation during surgery. Future AI must integrate multimodal data and adapt to micro- and nanorobotics.

**Table 2:** AI Applications in Molecular Profiling, Predictive Modeling, and Clinical Trial Integration.

Category	Details
AI in Molecular Profiling and Treatment Selection	AI algorithms analyze multi-OMICs data for molecular characterization, tumor grading, and clinical decision-making. Radiogenomics integrates imaging-derived parameters with genomic data. Radiomics extracts quantitative features from medical images to predict treatment response and patient outcomes. AI platforms optimize clinical trial protocols and patient recruitment. Examples include radiomic features predicting RAS mutation status in colorectal cancer, and radiogenomics identifying EGFR expression in lung cancer.
Predictive Modeling for Drug Response and Personalized Therapy	AI predicts tumor response to treatments, aiding personalized therapy. Radiomic signatures can predict response to treatments like FOLFIRI and detect EGFR-resistant tumors. In NSCLC, AI evaluates treatment efficacy and predicts outcomes, aiding in immunotherapy selection. Radiomic features from MRI can predict recurrence-free survival in breast cancer patients undergoing chemotherapy. AI can identify immune phenotypes in NSCLC, predicting response to immune checkpoint inhibitors.
Integrating AI in Clinical Trial Design and Patient Recruitment	AI improves clinical trial design and patient recruitment. Natural language processing (NLP) software analyzes large datasets to optimize trial protocols. AI predicts progression-free survival and overall survival in clinical trials, potentially replacing control arms with virtual arms. AI enhances patient recruitment by matching patients with suitable trials based on molecular profiles. AI platforms, like Watson for Clinical Trial Matching, have increased patient accrual in trials. Challenges include data standardization, reproducibility, and regulatory frameworks for health data.

**Table 3:** AI Applications in Radiotherapy Workflow and Outcome Prediction.

Category	Details
AI-enhanced Radiotherapy Workflow	AI, particularly deep learning, enhances contouring, treatment planning, optimization, and adaptive workflows. AI-based tools like the Radiation Planning Assistant (RPA) automate key components of radiotherapy planning, improving efficiency and accessibility, especially in low- and middle-income countries (LMICs). AI can automate repetitive tasks, optimize time, and improve clinical outcomes through predictive models and decision support systems (DSS). AI aids in all steps of radiotherapy, from patient consultation to treatment delivery, reducing clinical workload and improving quality assurance.
Prediction of Radiotherapy Outcomes and Toxicity	AI and machine learning (ML) predict radiotherapy outcomes and toxicity by analyzing complex medical data. Radiomics extract quantitative features from medical images, improving decision-making in precision medicine. AI models enhance prediction of side effects, integrating radiomic features, genetic factors, and imaging analyses. AI can predict treatment outcomes and toxicity, contributing to more personalized and accurate radiotherapy. Challenges include interpretability, validation, and standardization of AI models.
Addressing Uncertainties and Limitations in AI for Radiation Oncology	AI in radiation oncology faces challenges like lack of standardized protocols, small datasets, and need for regular model updates. Extensive clinical trials and standardized protocols are essential for effective integration of AI in clinical settings. Human intervention remains crucial to ensure quality and safety. Despite potential, AI’s full realization requires addressing uncertainties and constraints, ensuring patient safety, and efficacy in oncological radiotherapy.

**Table 4:** AI Integration in Multidisciplinary Tumor Boards (MTBs).

Category	Details
Enhancing Efficiency and Effectiveness	AI can analyze large datasets, including genomic, imaging, and clinical records, to identify patterns and correlations, providing valuable insights into tumor characteristics, treatment responses, and patient prognoses. This capability allows for more informed decision-making in cancer care.
Multidisciplinary Teams (MDTs)	MDTs, comprising medical professionals from various specialties, collaborate to define treatment plans for patients. AI-driven Clinical Decision Support Systems (CDSS) can reduce the time spent evaluating evidence-based practices, integrating clinical, imaging, biological, genetic, and cost-related data to produce predictive models.
Streamlining Administrative Tasks	AI can automate the collection and organization of patient data, reducing manual data entry and allowing clinicians to focus on critical case discussions. This improves the efficiency of the Multidisciplinary Tumor Board (MTB).
AI Tools in MTB	IBM’s Watson for Oncology, now equipped with advanced generative AI capabilities, has shown high concordance with MTB decisions and facilitates a multidisciplinary approach, saving time for simpler cases. Other AI tools and chatbots like ChatGPT are being explored to standardize language and interpretation of MTB discussions.
Optimizing Decision-Making	AI models, such as those developed by Kasprzak et al., can optimize decisions like whether molecular profiling should be performed, impacting patient outcomes.
Overall Impact of AI	Integrating AI in MTB can enhance diagnostic precision, personalize treatment plans, predict patient outcomes, and streamline administrative tasks, leading to more comprehensive, timely, and effective cancer care, and ultimately improving patient outcomes and advancing oncology.

## References

[1] Engelhardt M., Ihorst G., Schumacher M., Rassner M., Gengenbach L., Möller M., Shoumariyeh K., Neubauer J., Farthmann J., Herget G. (2021). Multidisciplinary Tumor Boards and Their Analyses: The Yin and Yang of Outcome Measures. BMC Cancer.

[2] El Saghir N.S., Keating N.L., Carlson R.W., Khoury K.E., Fallowfield L. (2014). Tumor Boards: Optimizing the Structure and Improving Efficiency of Multidisciplinary Management of Patients with Cancer Worldwide. Am. Soc. Clin. Oncol. Educ. Book.

[3] Basta Y.L., Baur O.L., van Dieren S., Klinkenbijl J.H., Fockens P., Tytgat K.M. (2016). Is There a Benefit of Multidisciplinary Cancer Team Meetings for Patients with Gastrointestinal Malignancies?. Ann. Surg. Oncol..

[4] Winters D.A., Soukup T., Sevdalis N., Green J.S.A., Lamb B.W. (2021). The Cancer Multidisciplinary Team Meeting: In Need of Change? History, Challenges and Future Perspectives. BJU Int..

[5] Ramesh A.N., Kambhampati C., Monson J.R., Drew P.J. (2004). Artificial Intelligence in Medicine. Ann. R. Coll. Surg. Engl..

[6] Lee K., Lee S.H. (2020). Artificial Intelligence-Driven Oncology Clinical Decision Support System for Multidisciplinary Teams. Sensors.

[7] Walsh S., de Jong E.E., van Timmeren J.E., Ibrahim A., Compter I., Peerlings J., Sanduleanu S., Refaee T., Keek S., Larue R.T. (2019). Decision Support Systems in Oncology. JCO Clin. Cancer Inform..

[8] Nagendran M., Chen Y., A Lovejoy C., Gordon A.C., Komorowski M., Harvey H., Topol E.J., A Ioannidis J.P., Collins G.S., Maruthappu M. (2020). Artificial Intelligence versus Clinicians: Systematic Review of Design, Reporting Standards, and Claims of Deep Learning Studies. BMJ.

[9] Topol E.J. (2019). High-Performance Medicine: The Convergence of Human and Artificial Intelligence. Nat. Med..

[10] Hashimoto D.A., Rosman G., Rus D., Meireles O.R. (2018). Artificial Intelligence in Surgery: Promises and Perils. Ann. Surg..

[11] Navarrete-Welton A.J., Hashimoto D.A. (2020). Current Applications of Artificial Intelligence for Intraoperative Decision Support in Surgery. Front. Med..

[12] Scally C.P., Varban O.A., Carlin A.M., Birkmeyer J.D., Dimick J.B. (2016). Video Ratings of Surgical Skill and Late Outcomes of Bariatric Surgery. JAMA Surg..

[13] Birkmeyer J.D., Finks J.F., O’Reilly A., Oerline M., Carlin A.M., Nunn A.R., Dimick J., Banerjee M., Birkmeyer N.J. (2013). Surgical Skill and Complication Rates after Bariatric Surgery. N. Engl. J. Med..

[14] O’Shea P. (2012). Future Medicine Shaped by an Interdisciplinary New Biology. Lancet.

[15] Ward T.M., Mascagni P., Madani A., Padoy N., Perretta S., Hashimoto D.A. (2021). Surgical Data Science and Artificial Intelligence for Surgical Education. J. Surg. Oncol..

[16] Baker G.R., Norton P.G., Flintoft V., Blais R., Brown A., Cox J., Etchells E., Ghali W.A., Hébert P., Majumdar S.R. (2004). The Canadian Adverse Events Study: The Incidence of Adverse Events among Hospital Patients in Canada. CMAJ.

[17] Brennan T.A., Leape L.L., Laird N.M., Hebert L., Localio A.R., Lawthers A.G., Newhouse J.P., Weiler P.C., Hiatt H.H. (1991). Incidence of Adverse Events and Negligence in Hospitalized Patients. Results of the Harvard Medical Practice Study I. N. Engl. J. Med..

[18] Forster A.J., Asmis T.R., Clark H.D., Al Saied G., Code C.C., Caughey S.C., Baker K., Watters J., Worthington J., van Walraven C. (2004). Ottawa Hospital Patient Safety Study: Incidence and Timing of Adverse Events in Patients Admitted to a Canadian Teaching Hospital. CMAJ.

[19] Gawande A.A., Thomas E.J., Zinner M.J., Brennan T.A. (1999). The Incidence and Nature of Surgical Adverse Events in Colorado and Utah in 1992. Surgery.

[20] Madani A., Vassiliou M.C., Watanabe Y., Al-Halabi B., Al-Rowais M.S., Deckelbaum D.L., Fried G.M., Feldman L.S. (2017). What Are the Principles That Guide Behaviors in the Operating Room?: Creating a Framework to Define and Measure Performance. Ann. Surg..

[21] Figura N., Marano L., Moretti E., Ponzetto A. (2016). Helicobacter Pylori Infection and Gastric Carcinoma: Not All the Strains and Patients Are Alike. World J. Gastrointest. Oncol..

[22] Kowalczyk O., Rypel A. (2023). Communicative Competence in Healthcare and Linguistic Theories: Insights and Applications. Acta Elbingensia.

[23] Januszko-Giergielewicz B., Wójcik-Kula A., Perliński J. (2023). Dynamic Changes in Teaching and Learning Methods in the Fild of Study of Medicine—Evolution, Not Revolution. Acta Elbingensia.

[24] Way L.W., Stewart L., Gantert W., Liu K., Lee C.M., Whang K., Hunter J.G. (2003). Causes and Prevention of Laparoscopic Bile Duct Injuries: Analysis of 252 Cases from a Human Factors and Cognitive Psychology Perspective. Ann. Surg..

[25] Madani A., Grover K., Watanabe Y. (2020). Measuring and Teaching Intraoperative Decision-Making Using the Visual Concordance Test: Deliberate Practice of Advanced Cognitive Skills. JAMA Surg..

[26] Madani A., Namazi B., Altieri M.S., Hashimoto D.A., Rivera A.M., Pucher P.H., Navarrete-Welton A., Sankaranarayanan G., Brunt L.M., Okrainec A. (2022). Artificial Intelligence for Intraoperative Guidance: Using Semantic Segmentation to Identify Surgical Anatomy During Laparoscopic Cholecystectomy. Ann. Surg..

[27] Brunt L.M., Deziel D.J., Telem D.A., Strasberg S.M., Aggarwal R., Asbun H., Bonjer J., McDonald M., Alseidi A., Ujiki M. (2020). Safe Cholecystectomy Multi-Society Practice Guideline and State of the Art Consensus Conference on Prevention of Bile Duct Injury During Cholecystectomy. Ann. Surg..

[28] Madani A., Watanabe Y., Feldman L.S., Vassiliou M.C., Barkun J.S., Fried G.M., Aggarwal R. (2015). Expert Intraoperative Judgment and Decision-Making: Defining the Cognitive Competencies for Safe Laparoscopic Cholecystectomy. J. Am. Coll. Surg..

[29] Nijssen M.A., Schreinemakers J.M., Meyer Z., van der Schelling G.P., Crolla R.M., Rijken A.M. (2015). Complications After Laparoscopic Cholecystectomy: A Video Evaluation Study of Whether the Critical View of Safety Was Reached. World J. Surg..

[30] Stefanidis D., Chintalapudi N., Anderson-Montoya B., Oommen B., Tobben D., Pimentel M. (2017). How Often Do Surgeons Obtain the Critical View of Safety during Laparoscopic Cholecystectomy?. Surg. Endosc..

[31] Mascagni P., Vardazaryan A., Alapatt D., Urade T., Emre T., Fiorillo C., Pessaux P., Mutter D., Marescaux J., Costamagna G. (2022). Artificial Intelligence for Surgical Safety: Automatic Assessment of the Critical View of Safety in Laparoscopic Cholecystectomy Using Deep Learning. Ann. Surg..

[32] Zhou X.Y., Guo Y., Shen M., Yang G.Z. (2020). Application of Artificial Intelligence in Surgery. Front. Med..

[33] Litjens G., Kooi T., Bejnordi B.E., Setio A.A.A., Ciompi F., Ghafoorian M., van der Laak J.A.W.M., van Ginneken B., Sánchez C.I. (2017). A Survey on Deep Learning in Medical Image Analysis. Med. Image Anal..

[34] Khosravi P., Kazemi E., Imielinski M., Elemento O., Hajirasouliha I. (2018). Deep Convolutional Neural Networks Enable Discrimination of Heterogeneous Digital Pathology Images. EBioMedicine.

[35] Żydowicz W.M., Skokowski J., Marano L., Polom K. (2024). Current Trends and Beyond Conventional Approaches: Advancements in Breast Cancer Surgery through Three-Dimensional Imaging, Virtual Reality, Augmented Reality, and the Emerging Metaverse. J. Clin. Med..

[36] Meyer A., Zverinski D., Pfahringer B., Kempfert J., Kuehne T., Sündermann S.H., Stamm C., Hofmann T., Falk V., Eickhoff C. (2018). Machine Learning for Real-Time Prediction of Complications in Critical Care: A Retrospective Study. The Lancet. Respir. Med..

[37] Li X., Zhang S., Zhang Q., Wei X., Pan Y., Zhao J., Xin X., Qin C., Wang X., Li J. (2019). Diagnosis of Thyroid Cancer Using Deep Convolutional Neural Network Models Applied to Sonographic Images: A Retrospective, Multicohort, Diagnostic Study. Lancet Oncol..

[38] Xia J., Cai Z., Heidari A.A., Ye Y., Chen H., Pan Z. (2022). Enhanced Moth-Flame Optimizer with Quasi-Reflection and Refraction Learning with Application to Image Segmentation and Medical Diagnosis. Curr. Bioinform..

[39] Xu Z., Heidari A.A., Kuang F., Khalil A., Mafarja M., Zhang S., Chen H., Pan Z. (2023). Enhanced Gaussian Bare-Bones Grasshopper Optimization: Mitigating the Performance Concerns for Feature Selection. Expert Syst. Appl..

[40] Xia J., Zhang H., Li R., Chen H., Turabieh H., Mafarja M., Pan Z. (2021). Generalized Oppositional Moth Flame Optimization with Crossover Strategy: An Approach for Medical Diagnosis. J. Bionic Eng..

[41] Marano L., Ricci A., Savelli V., Verre L., Di Renzo L., Biccari E., Costantini G., Marrelli D., Roviello F. (2019). From Digital World to Real Life: A Robotic Approach to the Esophagogastric Junction with a 3D Printed Model. BMC Surg..

[42] Cool D., Downey D., Izawa J., Chin J., Fenster A. (2006). 3D Prostate Model Formation from Non-Parallel 2D Ultrasound Biopsy Images. Med. Image Anal..

[43] Zhou X.Y., Yang G.Z., Lee S.L. (2018). A Real-Time and Registration-Free Framework for Dynamic Shape Instantiation. Med. Image Anal..

[44] Mahmood F., Durr N.J. (2018). Deep Learning and Conditional Random Fields-Based Depth Estimation and Topographical Reconstruction from Conventional Endoscopy. Med. Image Anal..

[45] Turan M., Almalioglu Y., Araujo H., Konukoglu E., Sitti M. (2017). A Non-Rigid Map Fusion-Based Direct SLAM Method for Endoscopic Capsule Robots. Int. J. Intell. Robot. Appl..

[46] Mountney P., Yang G.Z. (2008). Soft Tissue Tracking for Minimally Invasive Surgery: Learning Local Deformation Online. Med. Image Comput. Comput. Assist. Interv..

[47] Ye M., Giannarou S., Meining A., Yang G.Z. (2016). Online Tracking and Retargeting with Applications to Optical Biopsy in Gastrointestinal Endoscopic Examinations. Med. Image Anal..

[48] Wang R., Zhang M., Meng X., Geng Z., Wang F.Y. (2018). 3-D Tracking for Augmented Reality Using Combined Region and Dense Cues in Endoscopic Surgery. IEEE J. Biomed. Health Inform..

[49] Bernhardt S., Nicolau S.A., Soler L., Doignon C. (2017). The Status of Augmented Reality in Laparoscopic Surgery as of 2016. Med. Image Anal..

[50] Wang J., Suenaga H., Hoshi K., Yang L., Kobayashi E., Sakuma I., Liao H. (2014). Augmented Reality Navigation with Automatic Marker-Free Image Registration Using 3-D Image Overlay for Dental Surgery. IEEE Trans. Biomed. Eng..

[51] Pratt P., Ives M., Lawton G., Simmons J., Radev N., Spyropoulou L., Amiras D. (2018). Through the HoloLens^TM^ Looking Glass: Augmented Reality for Extremity Reconstruction Surgery Using 3D Vascular Models with Perforating Vessels. Eur. Radiol. Exp..

[52] Zhang X., Wang J., Wang T., Ji X., Shen Y., Sun Z., Zhang X. (2019). A Markerless Automatic Deformable Registration Framework for Augmented Reality Navigation of Laparoscopy Partial Nephrectomy. Int. J. Comput. Assist. Radiol. Surg..

[53] Granata V., Fusco R., Avallone A., De Stefano A., Ottaiano A., Sbordone C., Brunese L., Izzo F., Petrillo A. (2021). Radiomics-Derived Data by Contrast Enhanced Magnetic Resonance in RAS Mutations Detection in Colorectal Liver Metastases. Cancers.

[54] Yang L., Dong D., Fang M., Zhu Y., Zang Y., Liu Z., Zhang H., Ying J., Zhao X., Tian J. (2018). Can CT-Based Radiomics Signature Predict KRAS/NRAS/BRAF Mutations in Colorectal Cancer?. Eur. Radiol..

[55] Dercle L., Lu L., Schwartz L.H., Qian M., Tejpar S., Eggleton P., Zhao B., Piessevaux H. (2020). Radiomics Response Signature for Identification of Metastatic Colorectal Cancer Sensitive to Therapies Targeting EGFR Pathway. J. Natl. Cancer Inst..

[56] Drukker K., Li H., Antropova N., Edwards A., Papaioannou J., Giger M.L. (2018). Most-Enhancing Tumor Volume by MRI Radiomics Predicts Recurrence-Free Survival ``early on’’ in Neoadjuvant Treatment of Breast Cancer. Cancer Imaging Off. Publ. Int. Cancer ImagingSoc..

[57] Wong C.H., Siah K.W., Lo A.W. (2019). Estimation of Clinical Trial Success Rates and Related Parameters. Biostatistics.

[58] Haddad T., Helgeson J.M., E Pomerleau K., Preininger A.M., Roebuck M.C., Dankwa-Mullan I., Jackson G.P., Goetz M.P. (2021). Accuracy of an Artificial Intelligence System for Cancer Clinical Trial Eligibility Screening: Retrospective Pilot Study. JMIR Med. Inform..

[59] Zhou M., Leung A., Echegaray S., Gentles A., Shrager J.B., Jensen K.C., Berry G.J., Plevritis S.K., Rubin D.L., Napel S. (2018). Non-Small Cell Lung Cancer Radiogenomics Map Identifies Relationships between Molecular and Imaging Phenotypes with Prognostic Implications. Radiology.

[60] Forghani R., Savadjiev P., Chatterjee A., Muthukrishnan N., Reinhold C., Forghani B. (2019). Radiomics and Artificial Intelligence for Biomarker and Prediction Model Development in Oncology. Comput. Struct. Biotechnol. J..

[61] Fraser M., Sabelnykova V.Y., Yamaguchi T.N., Heisler L.E., Livingstone J., Huang V., Shiah Y.-J., Yousif F., Lin X., Masella A.P. (2017). Genomic Hallmarks of Localized, Non-Indolent Prostate Cancer. Nature.

[62] McCann S.M., Jiang Y., Fan X., Wang J., Antic T., Prior F., VanderWeele D., Oto A. (2016). Quantitative Multiparametric MRI Features and PTEN Expression of Peripheral Zone Prostate Cancer: A Pilot Study. AJR Am. J. Roentgenol..

[63] Renard-Penna R., Cancel-Tassin G., Comperat E., Varinot J., Léon P., Roupret M., Mozer P., Vaessen C., Lucidarme O., Bitker M.-O. (2015). Multiparametric Magnetic Resonance Imaging Predicts Postoperative Pathology but Misses Aggressive Prostate Cancers as Assessed by Cell Cycle Progression Score. J. Urol..

[64] Fischer S., Tahoun M., Klaan B., Thierfelder K.M., Weber M.-A., Krause B.J., Hakenberg O., Fuellen G., Hamed M. (2019). A Radiogenomic Approach for Decoding Molecular Mechanisms Underlying Tumor Progression in Prostate Cancer. Cancers.

[65] Jiang L., You C., Xiao Y., Wang H., Su G.-H., Xia B.-Q., Zheng R.-C., Zhang D.-D., Jiang Y.-Z., Gu Y.-J. (2022). Radiogenomic Analysis Reveals Tumor Heterogeneity of Triple-Negative Breast Cancer. Cell Rep. Med..

[66] Bera K., Schalper K.A., Rimm D.L., Velcheti V., Madabhushi A. (2019). Artificial Intelligence in Digital Pathology—New Tools for Diagnosis and Precision Oncology. Nat. Rev. Clin. Oncol..

[67] Huang W., Randhawa R., Jain P., Hubbard S., Eickhoff J., Kummar S., Wilding G., Basu H., Roy R. (2022). A Novel Artificial Intelligence-Powered Method for Prediction of Early Recurrence of Prostate Cancer After Prostatectomy and Cancer Drivers. JCO Clin. Cancer Inform..

[68] Marmorino F., Faggioni L., Rossini D., Gabelloni M., Goddi A., Ferrer L., Conca V., Vargas J., Biagiarelli F., Daniel F. (2023). The Prognostic Value of Radiomic Features in Liver-Limited Metastatic Colorectal Cancer Patients from the TRIBE2 Study. Future Oncol..

[69] Shi L., He Y., Yuan Z., Benedict S., Valicenti R., Qiu J., Rong Y. (2018). Radiomics for Response and Outcome Assessment for Non-Small Cell Lung Cancer. Technol. Cancer Res. Treat..

[70] Khorrami M., Prasanna P., Gupta A. (2020). Changes in CT Radiomic Features Associated with Lymphocyte Distribution Predict Overall Survival and Response to Immunotherapy in Non-Small Cell Lung Cancer. Cancer Immunol. Res..

[71] Park S., Ock C.-Y., Kim H., Pereira S., Park S., Ma M., Choi S., Kim S., Shin S., Aum B.J. (2022). Artificial Intelligence-Powered Spatial Analysis of Tumor-Infiltrating Lymphocytes as Complementary Biomarker for Immune Checkpoint Inhibition in Non-Small-Cell Lung Cancer. J. Clin. Oncol..

[72] Askin S., Burkhalter D., Calado G., El Dakrouni S. (2023). Artificial Intelligence Applied to Clinical Trials: Opportunities and Challenges. Health Technol..

[73] Luchini C., Pea A., Scarpa A. (2022). Artificial Intelligence in Oncology: Current Applications and Future Perspectives. Br. J. Cancer.

[74] Woo M. (2019). An AI Boost for Clinical Trials. Nature.

[75] Sangari N., Qu Y. A Comparative Study on Machine Learning Algorithms for Predicting Breast Cancer Prognosis in Improving Clinical Trials. Proceedings of the 2020 International Conference on Computational Science and Computational Intelligence (CSCI).

[76] Schperberg A.V., Boichard A., Tsigelny I.F., Richard S.B., Kurzrock R. (2020). Machine Learning Model to Predict Oncologic Outcomes for Drugs in Randomized Clinical Trials. Int. J. Cancer.

[77] Kolla L., Gruber F.K., Khalid O., Hill C., Parikh R.B. (2021). The Case for AI-Driven Cancer Clinical Trials—The Efficacy Arm in Silico. Biochim. Biophys. Acta Rev. Cancer.

[78] Haddad T.C., Helgeson J., Pomerleau K., Makey M., Lombardo P., Coverdill S., Urman A., Rammage M., Goetz M.P., LaRusso N. (2018). Impact of a Cognitive Computing Clinical Trial Matching System in an Ambulatory Oncology Practice. J. Clin. Oncol..

[79] Feijoo F., Palopoli M., Bernstein J., Siddiqui S., Albright T.E. (2020). Key Indicators of Phase Transition for Clinical Trials through Machine Learning. Drug Discov. Today.

[80] Chen Z.H., Lin L., Wu C.F., Li C.F., Xu R.H., Sun Y. (2021). Artificial Intelligence for Assisting Cancer Diagnosis and Treatment in the Era of Precision Medicine. Cancer Commun..

[81] Huynh E., Hosny A., Guthier C., Bitterman D.S., Petit S.F., Haas-Kogan D.A., Kann B., Aerts H.J.W.L., Mak R.H. (2020). Artificial Intelligence in Radiation Oncology. Nat. Rev. Clin. Oncol..

[82] Mayerhoefer M.E., Materka A., Langs G., Häggström I., Szczypiński P., Gibbs P., Cook G. (2020). Introduction to Radiomics. J. Nucl. Med..

[83] Arimura H., Soufi M., Kamezawa H., Ninomiya K., Yamada M. (2019). Radiomics with Artificial Intelligence for Precision Medicine in Radiation Therapy. J. Radiat. Res..

[84] Dercle L., Henry T., Carré A., Paragios N., Deutsch E., Robert C. (2021). Reinventing Radiation Therapy with Machine Learning and Imaging Bio-Markers (Radiomics): State-of-the-Art, Challenges and Perspectives. Methods.

[85] Isaksson L.J., Pepa M., Zaffaroni M., Marvaso G., Alterio D., Volpe S., Corrao G., Augugliaro M., Starzyńska A., Leonardi M.C. (2020). Machine Learning-Based Models for Prediction of Toxicity Outcomes in Radiotherapy. Front. Oncol..

[86] Zomkowska E., Zakrzewska M., Pilewski D., Zajączkiewicz H. (2023). Assessment of Nervomuscle Coordination in the Act of Swallowing Using Dynamic Imaging Tests Performed by Cone Beam Computer Tomography. Acta Elbingensia.

[87] Bourbonne V., Da-Ano R., Jaouen V., Lucia F., Dissaux G., Bert J., Pradier O., Visvikis D., Hatt M., Schick U. (2021). Radiomics Analysis of 3D Dose Distributions to Predict Toxicity of Radiotherapy for Lung Cancer. Radiother. Oncol..

[88] Kerns S.L., Kundu S., Oh J.H., Singhal S.K., Janelsins M., Travis L.B., Deasy J.O., Janssens A.C.J., Ostrer H., Parliament M. (2015). The Prediction of Radiotherapy Toxicity Using Single Nucleotide Polymorphism-Based Models: A Step Toward Prevention. Semin. Radiat. Oncol..

[89] de Biase A., Sourlos N., van Ooijen P.M.A. (2022). Standardization of Artificial Intelligence Development in Radiotherapy. Semin. Radiat. Oncol..

[90] Vandewinckele L., Claessens M., Dinkla A., Brouwer C., Crijns W., Verellen D., van Elmpt W. (2020). Overview of Artificial Intelligence-Based Applications in Radiotherapy: Recommendations for Implementation and Quality Assurance. Radiother. Oncol..

[91] Wang C., Zhu X., Hong J.C., Zheng D. (2019). Artificial Intelligence in Radiotherapy Treatment Planning: Present and Future. Technol. Cancer Res. Treat..

[92] Feng M., Valdes G., Dixit N., Solberg T.D. (2018). Machine Learning in Radiation Oncology: Opportunities, Requirements, and Needs. Front. Oncol..

[93] Somashekhar S., Sepúlveda M.-J., Puglielli S., Norden A., Shortliffe E., Kumar C.R., Rauthan A., Kumar N.A., Patil P., Rhee K. (2018). Watson for Oncology and Breast Cancer Treatment Recommendations: Agreement with an Expert Multidisciplinary Tumor Board. Ann. Oncol..

[94] Zhao X., Zhang Y., Ma X., Chen Y., Xi J., Yin X., Kang H., Guan H., Dai Z., Liu D. (2020). Concordance between Treatment Recommendations Provided by IBM Watson for Oncology and a Multidisciplinary Tumor Board for Breast Cancer in China. Jpn. J. Clin. Oncol..

[95] Kim M.-S., Park H.-Y., Kho B.-G., Park C.-K., Oh I.-J., Kim Y.-C., Kim S., Yun J.-S., Song S.-Y., Na K.-J. (2020). Artificial Intelligence and Lung Cancer Treatment Decision: Agreement with Recommendation of Multidisciplinary Tumor Board. Transl. Lung Cancer Res..

[96] Le Thien M.A., Redjdal A., Bouaud J., Séroussi B. (2022). Using Machine Learning on Imbalanced Guideline Compliance Data to Optimize Multidisciplinary Tumour Board Decision Making for the Management of Breast Cancer Patients. Stud. Health Technol. Inform..

[97] Botsis T., Murray J., Leal A., Palsgrove D., Wang W., White J.R., Velculescu V.E., Anagnostou V., Johns Hopkins Molecular Tumor Board Investigators (2022). Natural Language Processing Approaches for Retrieval of Clinically Relevant Genomic Information in Cancer. Stud. Health Technol. Inform..

[98] Ng S.S.T., Oehring R., Ramasetti N., Roller R., Thomas P., Chen Y., Moosburner S., Winter A., Maurer M.-M., Auer T.A. (2023). Concordance of a Decision Algorithm and Multidisciplinary Team Meetings for Patients with Liver Cancer-a Study Protocol for a Randomized Controlled Trial. Trials.

[99] Park Y.E., Chae H. (2024). The Fidelity of Artificial Intelligence to Multidisciplinary Tumor Board Recommendations for Patients with Gastric Cancer: A Retrospective Study. J. Gastrointest. Cancer.

[100] Lukac S., Dayan D., Fink V., Leinert E., Hartkopf A., Veselinovic K., Janni W., Rack B., Pfister K., Heitmeir B. (2023). Evaluating ChatGPT as an Adjunct for the Multidisciplinary Tumor Board Decision-Making in Primary Breast Cancer Cases. Arch. Gynecol. Obstet..

[101] Griewing S., Gremke N., Wagner U., Lingenfelder M., Kuhn S., Boekhoff J. (2023). Challenging ChatGPT 3.5 in Senology—An Assessment of Concordance with Breast Cancer Tumor Board Decision Making. J. Pers. Med..

[102] Griewing S., Knitza J., Boekhoff J., Hillen C., Lechner F., Wagner U., Wallwiener M., Kuhn S. (2024). Evolution of Publicly Available Large Language Models for Complex Decision-Making in Breast Cancer Care. Arch. Gynecol. Obstet..

[103] Kasprzak J., Westphalen C.B., Frey S., Schmitt Y., Heinemann V., Fey T., Nasseh D. (2024). Supporting the Decision to Perform Molecular Profiling for Cancer Patients Based on Routinely Collected Data through the Use of Machine Learning. Clin. Exp. Med..

[104] Williams S., Horsfall H.L., Funnell J.P., Hanrahan J.G., Khan D.Z., Muirhead W., Stoyanov D., Marcus H.J. (2021). Artificial Intelligence in Brain Tumour Surgery-An Emerging Paradigm. Cancers.

[105] Liao J., Li X., Gan Y., Han S., Rong P., Wang W., Li W., Zhou L. (2022). Artificial Intelligence Assists Precision Medicine in Cancer Treatment. Front. Oncol..

[106] McDougall R.J. (2019). Computer Knows Best? The Need for Value-Flexibility in Medical AI. J. Med. Ethics.

[107] Marano L., Fusario D., Savelli V., Marrelli D., Roviello F. (2021). Robotic versus Laparoscopic Gastrectomy for Gastric Cancer: An Umbrella Review of Systematic Reviews and Meta-Analyses. Updates Surg..

[108] Grunhut J. (2023). Artificial Intelligence: The Elephant in the Tumor Board Room. Acad. Med..

[109] Burr C., Leslie D. (2023). Ethical Assurance: A Practical Approach to the Responsible Design, Development, and Deployment of Data-Driven Technologies. AI Ethics.

[110] Burrell J. (2016). How the Machine ‘Thinks’: Understanding Opacity in Machine Learning Algorithms. Big Data Soc..

[111] Żydowicz W.M., Skokowski J., Marano L., Polom K. (2024). Navigating the Metaverse: A New Virtual Tool with Promising Real Benefits for Breast Cancer Patients. J. Clin. Med..

[112] Sap M., Card D., Gabriel S., Choi Y., Smith N.A. The Risk of Racial Bias in Hate Speech Detection. Proceedings of the ACL 2019—57th Annual Meeting of the Association for Computational Linguistics.

[113] Ahmet E. (2022). The Impact of Artificial Intelligence on Social Problems and Solutions: An Analysis on the Context of Digital Divide and Exploitation. Yeni Medya.

[114] Lee M.K. (2018). Understanding Perception of Algorithmic Decisions: Fairness, Trust, and Emotion in Response to Algorithmic Management. Big Data Soc..

[115] Selbst A.D., Boyd D., Friedler S.A., Venkatasubramanian S., Vertesi J. Fairness and Abstraction in Sociotechnical Systems. Proceedings of the FAT* 2019—Proceedings of the 2019 Conference on Fairness, Accountability, and Transparency.

[116] Ferrer X., Van Nuenen T., Such J.M., Cote M., Criado N. (2021). Bias and Discrimination in AI: A Cross-Disciplinary Perspective. IEEE Technol. Soc. Mag..

[117] Felzmann H., Fosch-Villaronga E., Lutz C., Tamò-Larrieux A. (2020). Towards Transparency by Design for Artificial Intelligence. Sci. Eng. Ethics.

[118] Schiff D., Rakova B., Ayesh A., Fanti A., Lennon M. (2020). Principles to Practices for Responsible AI: Closing the Gap. arXiv.

[119] Schmitt L. (2022). Mapping Global AI Governance: A Nascent Regime in a Fragmented Landscape. AI Ethics.

[120] Benjamins R., Rubio Viñuela Y., Alonso C. (2023). Social and Ethical Challenges of the Metaverse: Opening the Debate. AI Ethics.

[121] Habbal A., Ali M.K., Abuzaraida M.A. (2024). Artificial Intelligence Trust, Risk and Security Management (AI TRiSM): Frameworks, Applications, Challenges and Future Research Directions. Expert Syst. Appl..

[122] Kaissis G.A., Makowski M.R., Rückert D., Braren R.F. (2020). Secure, Privacy-Preserving and Federated Machine Learning in Medical Imaging. Nat. Mach. Intell..

[123] (2022). Information Security, Cybersecurity and Privacy Protection—Information Security Management Systems—Requirements.

[124] Dayan I., Roth H.R., Zhong A., Harouni A., Gentili A., Abidin A.Z., Liu A., Costa A.B., Wood B.J., Tsai C.S. (2021). Federated Learning for Predicting Clinical Outcomes in Patients with COVID-19. Nat. Med..

[125] Ghafur S., Grass E., Jennings N.R., Darzi A. (2019). The Challenges of Cybersecurity in Health Care: The UK National Health Service as a Case Study. Lancet Digit. Health.

